# The Potential Impact of Federal Funding Cuts on Access to Pre-Exposure Prophylaxis in Atlanta, Georgia: Geographic Modeling Study

**DOI:** 10.2196/89473

**Published:** 2026-02-13

**Authors:** Noah Mancuso, Patrick S Sullivan

**Affiliations:** 1 Department of Epidemiology Emory University Atlanta, GA United States; 2 Queer Health Collaborative Atlanta, GA United States

**Keywords:** HIV prevention, pre-exposure prophylaxis, community health centers, health services, geographic access, federal

## Abstract

**Background:**

Despite major biomedical advances in HIV testing, prevention, and treatment, annual HIV transmissions in the United States remain above 30,000. Geographic access to pre-exposure prophylaxis (PrEP) is critical to HIV prevention efforts, particularly in regions with high HIV burdens, such as metro-Atlanta. Community-based organizations (CBOs) play a central role in delivering culturally competent prevention services, yet many rely on federal funding that is increasingly unstable. Understanding the potential impact of CBO closures on geographic access to PrEP is essential for anticipating inequities and informing policy.

**Objective:**

The aim of this study was to estimate how hypothetical closures of federally funded CBOs providing PrEP affect geographic access to PrEP clinics by car and public transit across metro-Atlanta and to assess whether impacts differ by community racial/ethnic composition.

**Methods:**

We identified 71 PrEP-providing clinics in metro-Atlanta (August 2025), including 12 CBOs. Using 3 simulated closure scenarios in which 25% of CBOs were randomly closed, we calculated one-way travel times from 2466 census block group (CBG) centroids to the nearest PrEP-providing clinic. Travel times were estimated for car and public transit across 3 weekdays and timepoints and then averaged per CBG. Two-sided paired *t* tests were used to compare the change in travel time compared to baseline. Logistic regression assessed associations between racial/ethnic plurality and increased travel times.

**Results:**

Under baseline conditions, 100% of CBGs had car access to a PrEP clinic within 30 minutes compared to only 41.6% (1027/2466) via public transit. Across closure scenarios, 732 CBGs (29.6%; representing over 1 million residents) experienced increased transit times (mean increase 1.2 minutes; range 0.0-11.6; *P*<.001), and 7 CBGs lost transit access entirely. For car travel, 1184 CBGs (48%; representing approximately 1.7 million residents) experienced increased drive times (mean increase 0.5 minutes; range 0.0-6.4; *P*=.03). Black-plurality CBGs had higher odds of increased drive times compared to White-plurality CBGs (odds ratio 1.37, 95% CI 1.15-1.63).

**Conclusions:**

Even limited closure of CBO PrEP providers meaningfully reduces geographic access to HIV prevention services, disproportionately affecting communities already experiencing transportation and HIV-related vulnerabilities. Sustained federal investment in CBOs is essential to preserve equitable PrEP access and prevent avoidable HIV infections.

## Introduction

We have arrived at a point in the HIV epidemic in the United States where we arguably have all of the biomedical tools we need to end the epidemic [1]. There are multiple options for HIV screening, including self-testing [2]; multiple formulations and regimens for pre-exposure prophylaxis (PrEP) to prevent HIV infection; and effective treatments that result in full healthy lives for people living with HIV and, for people living with HIV who are virally suppressed, eliminating the risk of onward transmission through sex [3]. Despite these biomedical advances, annual HIV transmissions in the United States remain above 30,000 [4].

The realties of bringing these foundational tools to scale in a coordinated response has proven to be a substantial challenge of implementation and has resulted in increased attention to and funding for implementation science approaches [5-7]. Geographic access to HIV prevention services, including PrEP, is foundational to the “Prevent” pillar of the National HIV/AIDS Strategy [8]. We have previously explored geographic access to HIV prevention and care issues during a time of more certainty and consistency about the funding mechanisms for HIV prevention and care [9-11]. We have found that inequities in geographic access to HIV prevention and care services, and often, commute times disproportionately impact communities of color for whom car ownership may be less [12,13] and for whom public transportation access and route frequency may be more limited [14,15].

As we consider the structural and socioeconomic factors that impact geographic access to HIV prevention services, we are now in an era where federal funding for HIV services is declining or under threats of future reduction or elimination [16-19]. HIV prevention services in the United States are supported through a patchwork of federal funding mechanisms, including direct service delivery grants from the Centers for Disease Control and Prevention, funds administered through the Health Resources and Services Administration like the Ryan White HIV/AIDS Program, the Ending the HIV Epidemic initiative, Title X, Medicaid reimbursement for clinical PrEP services, and time-limited federal demonstration and implementation grants. These funding streams vary in stability, allowable services, and eligibility criteria, creating differential vulnerability to funding reductions across delivery settings. Although most PrEP prescriptions in the United States are provided through traditional health care clinics, community-based organizations (CBOs) contribute disproportionately to PrEP access among marginalized populations through direct provision, partnerships with prescribing clinicians, and intensive navigation services [20]. Given that many CBOs that provide HIV prevention services are supported, at least in part, by federal funding, it is important to consider how reductions in federal funding that lead to the closure of existing prevention providers might impact geographic access to HIV prevention services. Further, given the disparities in new HIV diagnoses related to acquisitions risks (eg, men who have sex with men), racial/ethnic minority groups (eg, Black and Hispanic or Latinx people) and geographic region (eg, the US South), it is important to assess whether gaps in geographic access to prevention services are differential by these population characteristics. To address these gaps, we used public data about the locations of HIV PrEP providers and previously described methods for estimating commute times to HIV prevention care services [10] to model the potential impact of federal funding cuts on geographic access to PrEP in Atlanta, Georgia.

## Methods

We examined how the closing of CBOs that provide HIV prevention services may impact access to PrEP by both car and public transit across the four counties that encompass metro-Atlanta: DeKalb, Cobb, Fulton, and Gwinnett (all of which are “Ending the HIV Epidemic”–prioritized counties for HIV prevention).

### Ethical Considerations

This study used publicly available, deidentified census and clinic location data. No human participants were involved, and therefore, no reviews or approvals by an ethics committee or institutional review board were required.

### Clinic Data and Closure Scenarios

We identified all PrEP-providing clinics in the metro-Atlanta area as of August 2025 by using the Centers for Disease Control and Prevention’s National Prevention Information Network PrEP Locator directory [21]. Clinics in the database are screened for eligibility if they have at least one health care professional (eg, physician, nurse practitioner, physician assistant) who is qualified to prescribe PrEP and if they have confirmed that they actively prescribe it. In addition to geographic data, each clinic is categorized by its funding type (federally qualified health center, public health department, hospital, CBO, etc). Of the 71 clinics in metro-Atlanta, 12 were designated as a CBO. To simulate potential impacts of federal funding reductions that disproportionately affect CBOs, we created 3 separate closure scenarios. In each scenario, 3 CBOs (approximately 25% of all Atlanta-area CBO PrEP clinics) were randomly selected for closure, and analyses were rerun to estimate the resulting changes in geographic access.

### Origin and Destination Specification

Census block groups (CBGs) served as the unit of analysis for travel time estimation. Origin coordinates were defined as the population-weighted centroid of each CBG, representing where residents are most likely to live. CBG-level sociodemographic data were obtained from the American Community Survey 5-year estimates [22]. CBGs were categorized by the racial or ethnic plurality of residents. Destination coordinates were the locations of PrEP-providing clinics identified above. This approach captures area-level variation in accessibility rather than individual travel patterns.

### Travel Time Estimation

We estimated one-way travel times between each CBG centroid and the 10 nearest PrEP clinics by using the Google Maps Distance Matrix application programming interface accessed through R [9]. The application programming interface was used to calculate the shortest available route under two transportation modes: public transit and private vehicle. For both modes, travel times were calculated across 3 weekdays (Tuesday, Friday, and Saturday) and 3 time points (8 AM, noon, and 3:30 PM).

The shortest one-way travel time for each day-time combination was selected and then averaged to provide one estimate per mode for each CBG for each closure scenario. CBGs with no available public transit route to any PrEP clinic, defined as a walking distance of more than 30 minutes to the nearest transit stop, were coded as having no transit access. Transit time estimates included walking time to any transit stops, waiting time, boarding/transfer time, and time in transit.

### Statistical Analysis

We used descriptive statistics to describe mean one-way travel times to the nearest PrEP clinic before and after simulated closures, separately for car and public transit. The mean change in travel time (minutes) was averaged across the 3 closure scenarios and compared using a 2-sided paired *t* test. CBGs were dichotomized into those that saw increased travel times compared to no change for both car and public transit. We then used simple logistic regression to model associations between race/ethnicity and increased travel times. All analyses and visualizations were conducted in R software (version 4.3.2; R Foundation for Statistical Computing).

## Results

### Descriptive Results

A total of 2466 CBGs across metro-Atlanta were included in the analysis. More than half of the CBGs (n=1361, 55.2%) had a White plurality. An additional 943 (38.2%) CBGs had a Black plurality, 155 (6.3%) had a Hispanic/Latinx plurality, and only 2 (<1%) CBGs had a plurality of another race or ethnicity. Under baseline conditions, all CBGs had access to at least one PrEP-providing clinic by car within 30 minutes, while only 1027 (41.6%) CBGs had access via public transit within 30 minutes. A total of 567 (22.9%) CBGs did not have access to PrEP via public transit.

### Public Transit Changes in Access

Across the 3 simulated CBO closure scenarios (N=2466), 732 (29.6%) CBGs experienced longer average public transit times to the nearest PrEP clinic compared with current access. These CBGs represented approximately 1,024,900 residents or about 27.8% of Atlanta’s total population. The average change in one-way transit time was 1.2 (range 0.0-11.6) minutes, which was significantly longer than the baseline (*P*<.001). Seven CBGs lost access to a PrEP clinic or CBO via public transit in the modeled closure scenarios. Compared to CBGs with a White plurality, the odds of experiencing increased transit times under the CBO closure scenarios were significantly lower for CBGs with a Hispanic plurality (odds ratio [OR] 0.61, 95% CI 0.40-0.95) but no different for CBGs with a Black plurality (OR 0.99, 95% CI 0.81-1.20).

### Drive Time Changes in Access

Across the 3 simulated CBO closure scenarios (N=2466), 1184 (48%) CBGs experienced longer average drive times to the nearest PrEP clinic compared with current access. These CBGs represented approximately 1,698,000 residents or about 46.4% of Atlanta’s total population. The average change in one-way drive time was 0.5 (range 0.0-6.4) minutes, which was significantly longer than baseline (*P*=.03). Compared to CBGs with a White plurality, the odds of experiencing increased drive times under the CBO closure scenarios were significantly higher for CBGs with a Black plurality (OR 1.37, 95% CI 1.15-1.63) but no different for CBGs with a Hispanic plurality (OR 0.90, 95% CI 0.64-1.28).

## Discussion

Our research explores the potential impact of federal funding cuts on geographic access to PrEP in Atlanta, Georgia. Whereas previous research in this area was proposed to identify geographic areas within cities that would be important locational targets for new services to decrease travel times to care [23-25], we argue that this expansion-oriented framing must be complemented by models of the closing of existing facilities due to cuts in HIV prevention funding to better understand PrEP service availability and the risk of new HIV infections. Our data suggest that increases in travel times related to the closure of community providers were greater for people who used public transit, underscoring the importance of evaluating modal dimensions of transportation time to health care facilities. This is especially relevant given that communities most vulnerable to HIV are also most likely to rely on public transportation [12,26].

Although the differences in travel times might seem modest in some settings, such differences represent meaningful barriers to seeking care among marginalized groups already facing transportation disadvantages. A given level of change in commute time may result in different willingness to travel for people who use different modes of transportation or for people who do not have employment benefits that include paid time for seeking health care [26,27]. It is also important to acknowledge that HIV prevention service providers have both located their facilities in areas with high needs for prevention services, and clinics that provide services in areas with fewer community and economic resources are often set up to meet multiple care and social service needs [28]. Colocation of PrEP services with other health and social services may facilitate engagement in prevention by reducing logistical barriers, normalizing HIV prevention within broader care settings, and leveraging established trust between providers and communities. Such organizations spend decades building trusting relationships with communities, fostering trusting relationships [29,30]. Depending on which facilities close, it is foreseeable that populations with multiple medical and social service needs might lose access to a variety of services that would increase their vulnerability to HIV and other health threats.

Other aspects of our analysis reinforce the stakes in terms of health and equity considerations that would attend closures of existing PrEP-prescribing organizations. Although any licensed medical provider can provide PrEP, CBOs are disproportionately located in high-need areas and serve populations often excluded from traditional health care systems [31]. Thus, the loss of such facilities would create a double impact on PrEP accessibility: it might create a longer commute time to the nearest PrEP provider, and it might mean that the nearest PrEP provider would be less likely to have culturally competent services [32-34]. Further, it is unclear whether existing clinics that survive the closure would have sufficient clinical capacity to provide PrEP to more patients even if prospective PrEP users are able to commute longer distances to continue to access PrEP [35,36]. Lastly, while our analysis focused on neighborhood level demographics, we know that PrEP-seeking patterns differ across multiple identity groups. For example, White men who have sex with men are more likely to access PrEP though traditional health care systems [37], while Black cisgender women and other groups with high HIV transmission tend to rely more often on community-based and safety-net providers [38]. Therefore, the loss of CBO-based PrEP services may disproportionately impact more marginalized groups.

The eventual impact of closures on the coverage of PrEP and the impacts of limitations on PrEP services are not easy to predict with confidence. However, it is clear that there are limitations to how far people will drive to obtain services for nonemergent health problems (eg, preventive services); several publications suggest that 30 minutes is a common threshold of willingness [39,40]. In any case, distance to receipt of care has an important and converse relationships with receipt of nonurgent health care, including preventive care [41]. A recent analysis suggests that even small decreases in PrEP coverage among people with indications could have substantial impacts on new HIV infections and health care costs. For example, just a 3% reduction in PrEP coverage is forecast to result in over 8000 preventable infections, with lifetime medical costs for HIV care exceeding US $3.6 billion for those infections [42].

Our findings, while empirically derived and reflective of real-world challenges to transportation to PrEP care services, have several limitations. First, we present one-way estimates of transit times, so the actual additional transportation burden to seeking PrEP care would be double the one-way estimates presented in the analysis. Simulated closures may not reflect actual likelihoods of closure; in the absence of empiric, publicly accessible data about organization-specific funding portfolios, we were unable to weight closures by likelihood and instead selected organizations randomly in the simulation. Future work incorporating such data could identify especially high-risk providers and communities. Conversely, we did not consider the likelihood of other types of organizations that provide PrEP closing; so, our estimates of impact on PrEP service might not reflect the true additional travel burden associated with the closure of facilities. Further, our analysis did not collect new data on, or consider the service capacity of, clinics; so, even if potential PrEP users were willing to travel longer distances, it is possible that existing clinics might not be able to handle an increase in the patient load.

According to our data, the closure of even a small number of current PrEP providers would have important impacts on physical access to HIV prevention services and would be predicted to increase commute times, decrease engagement in PrEP care, and result in avoidable new HIV infections and associated care costs. Therefore, our findings support sustained investment in CBOs as critical access points for HIV prevention services.

## Abbreviations

CBG: census block group
CBO: community-based organization
OR: odds ratio
PrEP: pre-exposure prophylaxis

## References

[1] Eisinger RW, Fauci AS (2018). Ending the HIV/AIDS pandemic. Emerg Infect Dis.

[2] Hecht J, Sanchez T, Sullivan PS, DiNenno EA, Cramer N, Delaney KP (2021). Increasing access to HIV testing through direct-to-consumer HIV self-test distribution - United States, March 31, 2020-March 30, 2021. MMWR Morb Mortal Wkly Rep.

[3] Cohen MS, Chen YQ, McCauley M, Gamble T, Hosseinipour MC, Kumarasamy N, Hakim JG, Kumwenda J, Grinsztejn B, Pilotto JHS, Godbole SV, Chariyalertsak S, Santos BR, Mayer KH, Hoffman IF, Eshleman SH, Piwowar-Manning E, Cottle L, Zhang XC, Makhema J, Mills LA, Panchia R, Faesen S, Eron J, Gallant J, Havlir D, Swindells S, Elharrar V, Burns D, Taha TE, Nielsen-Saines K, Celentano DD, Essex M, Hudelson SE, Redd AD, Fleming TR, HPTN 052 Study Team (2016). Antiretroviral therapy for the prevention of HIV-1 transmission. N Engl J Med.

[4] (2024). HIV surveillance supplemental report: estimated HIV incidence and prevalence in the United States, 2018-2022. Centers for Disease Control and Prevention.

[5] Parker RG, Easton D, Klein CH (2000). Structural barriers and facilitators in HIV prevention: a review of international research. AIDS.

[6] Beyrer C, Adimora AA, Hodder SL, Hopkins E, Millett G, Mon SHH, Sullivan PS, Walensky RP, Pozniak A, Warren M, Richman B, Copeland R, Mayer KH (2021). Call to action: how can the US Ending the HIV Epidemic initiative succeed?. The Lancet.

[7] Proctor EK, Bunger AC, Lengnick-Hall R, Gerke DR, Martin JK, Phillips RJ, Swanson JC (2023). Ten years of implementation outcomes research: a scoping review. Implement Sci.

[8] Updated to 2020 - indicator supplement (2016). National HIV/AIDS Strategy (NHAS).

[9] Mancuso N, Sullivan PS (2025). Methods for estimating public transit travel times to healthcare services as a measure of equitable healthcare access. Ann Epidemiol.

[10] Mancuso N, Weiss K, Siegler AJ, Sullivan PS (2026). Changes in geographic drive time access to pre-exposure prophylaxis (PrEP) in the United States, 2017 to 2025. Prev Med Rep.

[11] Luan H, Ransome Y, Taggart T, Chow J, Mancuso N, Grant M, Huang P, Sullivan P (2025). A comparison of HIV pre-exposure prophylaxis accessibility by public transit- and drive-time in Dallas-Fort Worth, Texas, 2024. Health Place.

[12] Dasgupta S, Kramer MR, Rosenberg ES, Sanchez TH, Reed L, Sullivan PS (2015). The effect of commuting patterns on HIV care attendance among men who have sex with men (MSM) in Atlanta, Georgia. JMIR Public Health Surveill.

[13] Goswami Neela D, Schmitz Michelle M, Sanchez Travis, Dasgupta Sharoda, Sullivan Patrick, Cooper Hannah, Rane Deepali, Kelly Jane, Del Rio Carlos, Waller Lance A (2016). Understanding local spatial variation along the care continuum: the potential impact of transportation vulnerability on hiv linkage to care and viral suppression in high-poverty areas, Atlanta, Georgia. J Acquir Immune Defic Syndr.

[14] Anderson KF, Galaskiewicz J (2021). Racial/ethnic residential segregation, socioeconomic inequality, and job accessibility by public transportation networks in the United States. Spat Demogr.

[15] Borowski E, Ermagun A, Levinson D (2019). Disparity of access: variations in transit service by race, ethnicity, income, and auto availability. https://trid.trb.org/View/1572981.

[16] Chan PA, Nunn AS (2025). Grant terminations in the US threaten HIV research and future progress. JAMA.

[17] Elendu Chukwuka, Amaechi Dependable C, Elendu Tochi C, Amaechi Emmanuel C, Elendu Ijeoma D, Akpa Kenneth N, Oloyede Praise O, Adegbola Michael O, Idowu Omoyelemi F (2025). Shaping sustainable paths for HIV/AIDS funding: a review and reminder. Ann Med Surg (Lond).

[18] McClure M, Gandhi M (2025). Threat of HIV and tuberculosis drug resistance after US funding cuts. The Lancet Infectious Diseases.

[19] Moore Christopher, Berl Tyler (2025). Sustaining the fight: maintaining HIV service funding in Delaware. Dela J Public Health.

[20] Smith DK, Maier E, Betts J, Gray S, Kolodziejski B, Hoover KW (2016). What community-based HIV prevention organizations say about their role in biomedical HIV prevention. AIDS Educ Prev.

[21] Siegler AJ, Wirtz S, Weber S, Sullivan PS (2017). Developing a web-based geolocated directory of HIV pre-exposure prophylaxis-providing clinics: The PrEP locator protocol and operating procedures. JMIR Public Health Surveill.

[22] American community survey 2016-2020 5-year data release. US Census Bureau.

[23] Harrington KRV, Hamilton C, Alohan DI, Hudson A, Young HN, Crawford ND (2025). The PrEP pharmacy reach study: protocol for the creation of maps to visualize the impact of expanding access to HIV prevention services through pharmacies. JMIR Public Health Surveill.

[24] Aral SO, Torrone E, Bernstein K (2015). Geographical targeting to improve progression through the sexually transmitted infection/HIV treatment continua in different populations. Curr Opin HIV AIDS.

[25] Kedziora DJ, Stuart RM, Pearson J, Latypov A, Dierst-Davies R, Duda M, Avaliani N, Wilson DP, Kerr CC (2019). Optimal allocation of HIV resources among geographical regions. BMC Public Health.

[26] Kabayundo J, Ahuja M, Jadhav S, Smith LC, Bithi FA, Shahi S, Johnson G, Munde S, Ratnapradipa KL (2025). Transportation barriers to healthcare access: a scoping review of measurement approaches and associated health outcomes. Transportation Research Interdisciplinary Perspectives.

[27] Harrison SE, Hung P, Green K, Miller SJ, Paton M, Ahuja D, Weissman S, Rudisill C, Evans T (2025). Does travel time matter?: predictors of transportation vulnerability and access to HIV care among people living with HIV in South Carolina. BMC Public Health.

[28] Robillard AG, Julious CH, Smallwood SW, Douglas M, Gaddist BW, Singleton T (2022). Structural inequities, HIV community-based organizations, and the end of the HIV epidemic. Am J Public Health.

[29] Elkins JS, Dale I, Okumu E, Zinck M, Golin C, McCrimmon J, Esposito M, Duguid IT, Craven DM, Onyeama U, Marin-Cespedes S, Diggs A, Indyg M, Dennis AM, Zarwell M (2025). Survival, trust, and compassion: persistent HIV prevention concerns faced by Black sexual and gender minority communities in the US South - a qualitative study. Front Public Health.

[30] Corbie-Smith G, Isler MR, Miles MS, Banks B (2012). Community-based HIV clinical trials: an integrated approach in underserved, rural, minority communities. Prog Community Health Partnersh.

[31] Mantell Joanne E, Bauman Laurie J, Bonett Stephen, Buchbinder Susan, Hoffman Susie, Storholm Erik D, McCoy Katryna, Rael Christine T, Cowan Ethan, Gonzalez-Argoti Tatiana, Safa Hussein, Scott Hyman, Murtaugh Kimberly Ling, Wilson Natalie L, Liu Albert (2025). Innovation in Providing Equitable Pre-exposure Prophylaxis Services in the United States: Expanding Access in Nontraditional Settings. J Acquir Immune Defic Syndr.

[32] Gómez Walter, Gomez AM, Solis S, Dimonte C, Organista KC (2025). Provider perspectives on multi-level barriers and facilitators to PrEP access among Latinx sexual and gender minorities. J Racial Ethn Health Disparities.

[33] Ross J, Betancourt GS, Andrade EA, Klein A, Marrero L, Morales GA, Rivera S, Watnick DL, Patel VV (2023). Collaborative PrEP implementation strategies for Latino men who have sex with men: a health center-community consensus process. J Community Health.

[34] Self KJ, Johnson A, Craker L, Silvey R, Fallon S, Cunningham SR, Kanamori M (2025). Strengthening PrEP services at community-based organizations for Latinx men who have sex with men: an implementation science approach. Arch Public Health.

[35] Agovi AM, Anikpo I, Cvitanovich MJ, Craten KJ, Asuelime EO, Ojha RP (2020). Knowledge needs for implementing HIV pre-exposure prophylaxis among primary care providers in a safety-net health system. Prev Med Rep.

[36] Ard Kevin L, Uzoeghelu Ugochukwu, Bruno Jack, Lambert Cei, Mayer Kenneth H, Davis John A, Keuroghlian Alex S (2021). Readiness of US federally qualified health centers to provide HIV pre-exposure prophylaxis. Open Forum Infect Dis.

[37] Kanny D, Jeffries W, Chapin-Bardales J, Denning P, Cha S, Finlayson T, Wejnert C (2017). Racial/Ethnic Disparities in HIV Preexposure Prophylaxis Among Men Who Have Sex with Men — 23 Urban Areas. MMWR Morb Mortal Wkly Rep. Sep 20, 2019.

[38] Boudreaux J, Valdebenito CM, Pichon LC (2025). Identifying access barriers to PrEP among cisgender black/African American women in the United States: a systematic review of the literature. Healthcare (Basel).

[39] Yen W (2013). How long and how far do adults travel and will adults travel for primary care?. Washington State Office of Financial Management.

[40] Baldomero AK, Kunisaki KM, Wendt CH, Bangerter A, Diem SJ, Ensrud KE, Nelson DB, Henning-Smith C, Bart BA, Hammett P, Hagedorn HJ, Dudley RA (2022). Drive time and receipt of guideline-recommended screening, diagnosis, and treatment. JAMA Netw Open.

[41] Koob C, Babatunde A, Baxter SLK, Parisi M, Griffin SF (2025). Examining primary care utilization and distance traveled for care among Medicaid-insured children in South Carolina. J Community Health.

[42] Sullivan PS, Wall KM, Juhasz M, DuBose S, Crowley JS, Breyer C, Millett G, Brisco K, Le G, Mayer K (2025). Excess HIV infections and costs associated with reductions in HIV prevention services in the US. JAMA Netw Open.

